# Regulation of CHK1 by mTOR contributes to the evasion of DNA damage barrier of cancer cells

**DOI:** 10.1038/s41598-017-01729-w

**Published:** 2017-05-08

**Authors:** Xinhui Zhou, Weijin Liu, Xing Hu, Adrienne Dorrance, Ramiro Garzon, Peter J. Houghton, Changxian Shen

**Affiliations:** 1 Department of Gynecology, the First Affiliated Hospital, School of Medicine, Zhejiang University, Hangzhou, Zhejiang China; 2 College of Biology and Food Engineering, Huaihua University, Huaihua, Hunan China; 3 Comprehensive Cancer Center, The Ohio State University, Columbus, Ohio USA; 4 The Greehey Children’s Cancer Research Institute at the UT Health Science Center, San Antonio, Texas USA

## Abstract

Oncogenic transformation leads to dysregulated cell proliferation, nutrient deficiency, and hypoxia resulting in metabolic stress and increased DNA damage. In normal cells, such metabolic stress leads to inhibition of signaling through the mammalian Target of Rapamycin Complex 1 (mTORC1), reduction of protein translation, cell cycle arrest, and conservation of energy. In contrast, negative regulation of mTORC1 signaling by DNA damage is abrogated in many cancer cells, thus mTORC1 signaling remains active under microenvironmental conditions that potentially promote endogenous DNA damage. Here we report that mTORC1 signaling suppresses endogenous DNA damage and replication stress. Pharmacological inhibition of mTOR signaling resulted in phosphorylation of H2AX concomitant with the decrease of CHK1 levels both in cell culture and mouse rhadomyosarcoma xenografts. Further results demonstrated that mTORC1-S6K1 signaling controls transcription of *CHK1* via Rb-E2F by upregulating cyclin D and E. Consistent with these results, downregulation of CHK1 by inhibition of mTOR kinase resulted in defects in the slow S phase progression following DNA damage. These results indicate that, under stressful conditions, maintained mTORC1 signaling in cancer cells promotes survival by suppressing endogenous DNA damage, and may control cell fate through the regulation of CHK1.

## Introduction

To survive the constant attack from endogenous and exogenous genotoxins, all organisms have evolved genome surveillance systems (checkpoints)^1^. The ATM-CHK2 and ATR-CHK1 checkpoints are the central genome surveillance systems that function to maximize cell survival while minimizing genome instability^2^. Activated CHK2 and CHK1 phosphorylate numerous downstream effectors to amplify and relay the signals to engage the DNA damage response (DDR) such as cell cycle arrest, DNA damage repair, senescence or apoptosis^1, 3^. The major functions of DNA damage checkpoints are to facilitate DNA repair and promote recovery from replication block^4, 5^ thereby maintaining cell survival. DNA replication forks undergo frequent stalling during normal cell cycle progression when they encounter endogenous DNA lesions estimated to occur at a frequency of at least 2 × 10^4^ per cell/day^6^. From yeast to mammalian cells, stabilization of stalled replication forks is regulated by ATR-CHK1, which makes the ATR-CHK1 checkpoint essential for cell survival in all eukaryotes^3, 7^. Moreover, eukaryotes have a highly efficient DNA repair network; under normal growth conditions, the baseline DNA damage incurred from extracellular and intracellular agents will be rapidly repaired and there is no checkpoint activation. However, in response to massive DNA damage, DNA damage checkpoint will be activated to arrest cell cycle progression in order to provide time for repair machinery to repair DNA lesions. Concomitant with checkpoint activation, mammalian TOR Complex 1 (mTORC1) signaling is suppressed^8^. When DNA damage is irreparable, the activated checkpoint promotes cell death via apoptosis in higher eukaryotes. Thus, through checkpoint signaling genome integrity is maintained^1, 9^.

Cancerous cells are characterized by dysregulation of multiple intracellular signaling networks as a consequence of around 100 genetic and epigenetic changes in solid tumors^10, 11^. Oncogene activation triggers replication stress and DNA damage, thereby increasing genome instability, an enabling characteristic of cancer cells^12, 13^. Oncogene-induced DNA replication stress has been postulated to result from the accelerated proliferation rate of cancer cells^13^. Because of the transient and long-term lack of nutrients, oxygen, and growth factors, rapid proliferating cancer cells also undergo frequent metabolic stress, another hallmark of cancer cells^14^. Thus, most cancer cells demonstrate DNA damage stress and elevated spontaneous DNA damage response.

mTORC1 acts as a node integrating intracellular and extracellular signal transduction networks via sensing multiple signals, and regulates cell metabolism, proliferation and survival^15–18^. Mounting evidence demonstrates that deregulation of AKT-mTOR signaling leads to cancer^19^ and overexpression of eIF4E enhances tumor formation^20^. Metabolic stress, such as nutrient starvation, hypoxia or deprivation of growth factors, results in downregulation of mTORC1 signaling in normal cells^18, 21, 22^. However, in cancer cells negative regulation of mTORC1 by DNA damage^8^ or hypoxia^23^ is defective, either through inactivation of p53 or ATM signaling. Maintained mTORC1 signaling under conditions of stress would maintain protein translation, cell cycle progression, but at the expense of increased energy metabolism. Thus, potentially, maintained mTORC1 signaling could have deleterious effects. Yet in most cancers, control of mTORC1 under stress is dysregulated. It was thus intriguing to postulate that maintained mTORC1 signaling may prevent DNA damage, and promote cell survival under conditions of metabolic stress. In this study, using pediatric rhabdomyosarcoma models *in vitro* and *in vivo*, we found that mTORC1 signaling suppresses spontaneous DNA damage and replication stress by controlling CHK1.

## Results

### Inhibition of mTOR Signaling Results in Spontaneous DNA Damage

**Figure 1:**
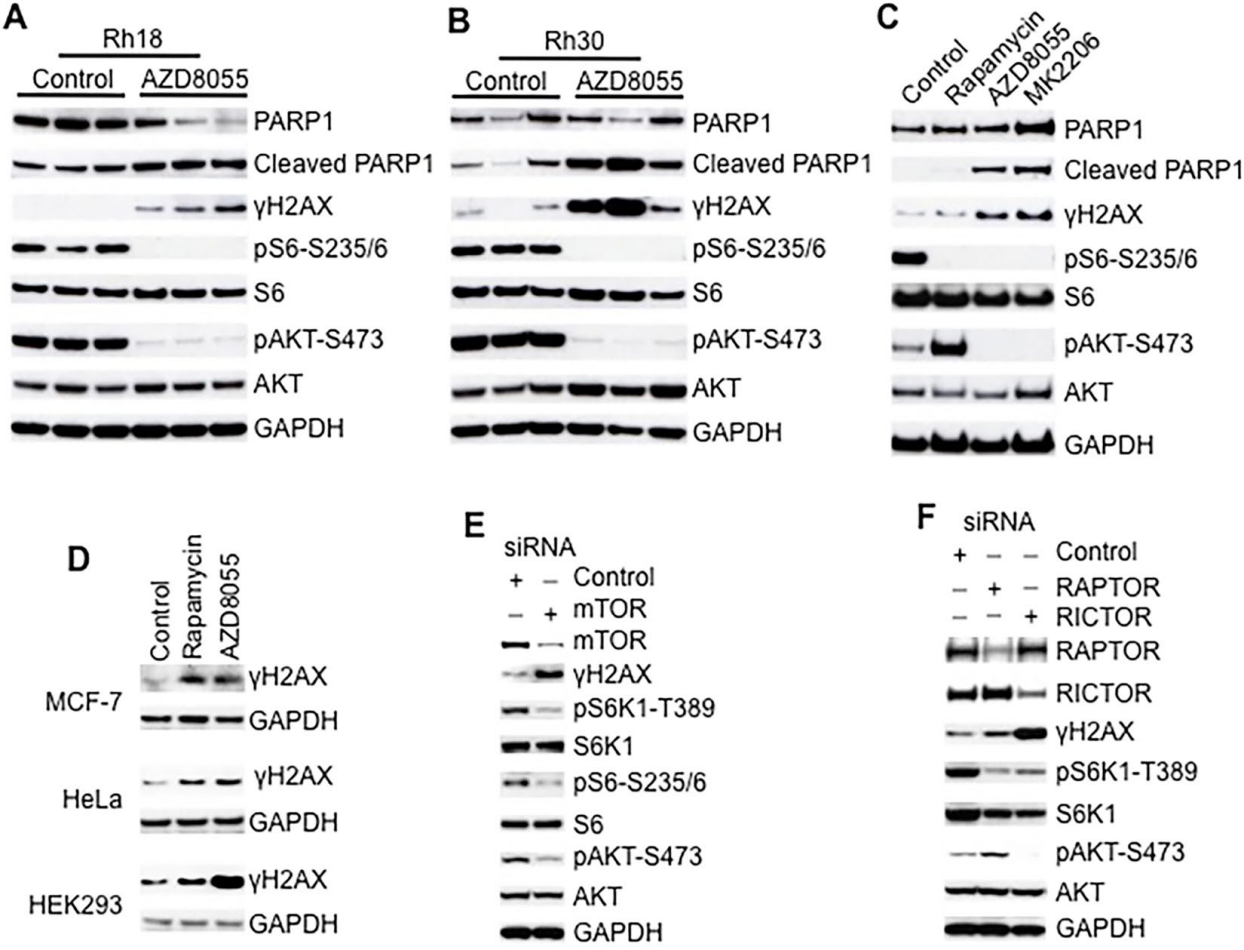
Inhibition of mTOR Signaling Results in DNA Damage. (**A**) Rh18 pediatric rhabdomyosarcoma tumor xenografts were propagated subcutaneously in SCID mice and were treated with mTOR kinase inhibitor AZD8055 at 20 mg/kg/day. Tumors were harvested 24 hr post treatment on day 4 and were pulverized under liquid N_2._ Total proteins were extracted for immunoblotting. Three tumors were randomly picked for each group. (**B**) Rh30 pediatric rhabdomyosarcoma tumor xenografts were manipulated and immunoblotting was done as in Fig. 1A. (**C**) Rh30 cells were treated with rapamycin (100 ng/mL), AZD8055 (1 μM) or MK2206 (10 μM) for 16 hr. Total proteins were extracted for immunoblotting as in Fig. 1A. (**D**), MCF-7, HeLa and HEK293 cells were treated with rapamycin (100 ng/mL) or AZD8055 (1 μM) for 16 hr. Total proteins were extracted for immunoblotting of γH2AX and GAPDH. (**E**) Rh30 cells were transfected with control or mTOR siRNA. 48 hr later, total proteins were extracted for immunoblotting. (**F**) Rh30 cells were transfected with control, RAPTOR or RICTOR siRNA. 48 hr later, total proteins were extracted for immunoblotting. GAPDH served as loading controls. Immunoblots were converted to white and black with auto tone by Photoshop program.

To examine the potential role of mTOR signaling in DNA damage response, we used an mTOR-selective kinase inhibitor rather than rapamycin, as in many cell lines rapamycin poorly inhibits 4E-BP1 phosphorylation^24^ and induces mTORC2 as a consequence of S6K1 inhibition leading to stabilization of IRS-1^15, 25^. Previous studies have demonstrated that AZD8055 is a potent and specific mTOR kinase inhibitor^26, 27^. Treatment of mice bearing rhabdomyosarcoma xenografts Rh18 (Fig. 1A) and Rh30 (Fig. 1B), with AZD8055 potently inhibited both mTORC1 and mTORC2 as demonstrated by the absence of pS6-S235/236 and pAKT-S473^27^. Unexpectedly, inhibition of mTOR kinase by AZD8055 led to obvious phosphorylation of H2AX (γH2AX), a marker of DNA damage, and elevated cleavage of PARP1, a marker of apoptosis (Fig. 1A and B). These results suggest that one function of mTOR kinase is to prevent spontaneous DNA damage and protect cells from apoptosis in these tumors.

To extend this observation *in vitro*, we treated cultured rhabdomyosarcoma Rh30 cells with rapamycin, AZD8055 or the AKT kinase inhibitor MK2206^28^. Rapamycin inhibited pS6-S235/236, a marker of mTORC1 activity, but increased pAKT-S473 probably due to inhibiting the mTORC1-S6K1-IRS negative feedback loop. Both AZD8055 and MK2206 inhibited pS6-S235/236 and pAKT-S473 signals. Similar to the effect in mouse xenografts, AZD8055 induced γH2AX and cleavage of PARP1 in Rh30 cells, as did the AKT inhibitor MK2206 (Fig. 1C). Rapamycin slightly induced γH2AX but not cleavage of PARP1 (Fig. 1C). In HEK293, MCF-7 and HeLa cells, both rapamycin and AZD8055 induced γH2AX (Fig. 1D). In addition, immunofluorescence microscope analysis demonstrated an increase of γH2AX staining in Rh30 cells treated with AZD8055 when compared to the untreated control (Supplementary Figure S1). Furthermore, analysis of metaphase chromosome by chromatin spreading assay showed that the number of radial chromosomes increased in Rh30 cells treated with either rapamycin or AZD8055 but with even higher number in AZD8055 treated cells (Supplementary Figure S2). To test the role of mTORC1 and mTORC2 in preventing endogenous DNA damage, we next downregulated mTOR, RAPTOR and RICTOR by siRNA. Knockdown of mTOR led to attenuation of pS6K1-T389, pS6-S235/236, pAKT-S473, and increased γH2AX (Fig. 1E). Knockdown of RICTOR also led to decreased pAKT-S473, pS6K1-T389, and elevation of γH2AX. In addition, knockdown of RAPTOR reduced pS6K1-T389, slightly increased the γH2AX and pAKT-S473 signals (Fig. 1F). These data support the idea that mTOR signaling suppresses spontaneous DNA damage of cancer cells both *in vitro* and *in vivo*.

### Inhibition of mTOR Signaling Impairs the CHK1 Checkpoint

**Figure 2:**
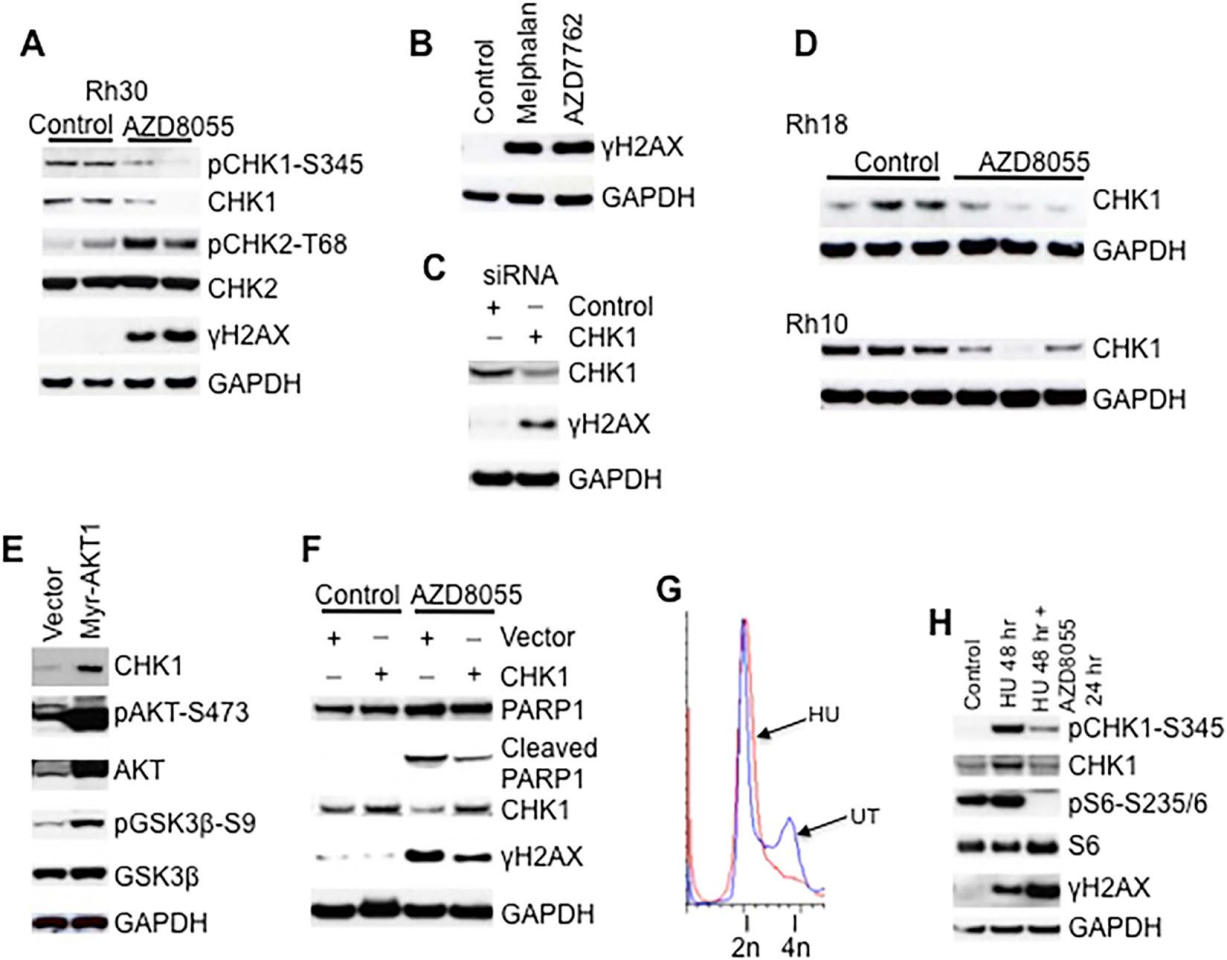
Inhibition of mTOR Signaling Impairs CHK1 DNA Replication Checkpoint. (**A**) Protein extracts of Rh30 tumor xenografts treated with vehicle or AZD8055 were used for immunoblotting. Two tumors were randomly picked for each group. (**B**) Rh30 cells were treated with CHK1 kinase inhibitor AZD7762 (1 μM) or melphalan (10 μg/mL) for 5 hr. Immunblotting was done to determine γH2AX. (**C**) Rh30 cells were transfected with control or CHK1 siRNA. 48 hr later, total proteins were extracted for immunoblotting of γH2AX and CHK1. (**D**), As in Fig. 2A, CHK1 antibody were used to determine the total protein levels of CHK1 in Rh18 and Rh10 tumor xenograft models treated with AZD8055. (**E**) Rh30 cells were transfected with vector or Myr-AKT1 plasmid. 48 hr later, total proteins were extracted for immunoblotting. (**F**) Rh30 cells were transfected with vector or CHK1 plasmid. 24 hr later, AZD8055 was added for additional 24 hr. Total proteins were extracted for immunoblotting. (**G**) Rh30 cells were treated with hydroxyurea (HU) at 2 mM for 24 hr to induce S-phase arrest. Flow cytometry was performed to show the early S phase arrest by HU and compared to untreated (UT) cells. 2n, diploid DNA; 4n, tetraploid DNA. (**H**) Rh30 cells were treated with HU at 2 mM for 24 hr, AZD8055 (1 μM) was added for additional 24 hr. pCHK1-S345, CHK1, S6, pS6-235/6 and γH2AX antibodies were applied for immunoblotting. Control, without treatment; HU 48 hr, cells treated for 48 hr; HU 48 hr + AZD8055 24 hr, cells treated with HU for 48 hr and AZD8055 for 24 hr in the presence of HU. GAPDH served as loading controls. Immunoblots were converted to gray with auto tone by Photoshop program.

Induction of γH2AX following inhibition of mTOR signaling indicates DNA strand breaks and suggests that mTOR signaling plays a role in DNA damage response (DDR). We thus examined the CHK1 and CHK2 checkpoint proteins, and found a decrease of both pCHK1-S345 and CHK1 in Rh30 xenografts treated with AZD8055. Decrease of CHK1 coincided with the elevation of the γH2AX signal (Fig. 2A). In contrast, AZD8055 increased pCHK2-T68 signal, indicators of ATM-CHK2 checkpoint activation (Fig. 2A). These results are consistent with our recent observation that mTORC1 signaling negatively regulates ATM in these tumors^29^. CHK1 is tightly controlled during the cell cycle progression and prevents DNA damage by stabilizing stalled DNA replication forks and promoting DNA repair. Indeed, in Rh30 cells inhibition of CHK1 kinase by AZD7762 resulted in γH2AX, to an extent similar to that induced by the DNA alkylator melphalan (Fig. 2B). Moreover, knockdown of CHK1 by siRNA also led to an increase in γH2AX signal (Fig. 2C). These data suggest that AZD8055-induced γH2AX might result from the impairment of the CHK1 checkpoint, at least in part, due to the decrease of CHK1. To examine whether similar effects were seen in other sarcoma models, we determined CHK1 levels in Rh18 and Rh10 tumor xenografts treated with AZD8055 and found that CHK1 was markedly downregulated by AZD8055 (Fig. 2D), a result similar to that of Rh30 xenografts (Fig. 2A). Consistent with mTOR regulation of CHK1, expression of myr-AKT1 increased CHK1 in Rh30 cells, accompanied with increased pAKT-S473 and pGSK3β-S9 (Fig. 2E). These data, taken together, indicate that CHK1 is regulated by mTOR both *in vivo* and *in vitro*.

To further examine whether γH2AX induction by inhibition of mTOR signaling results from the downregulation of CHK1, we transfected Rh30 cells with *CHK1* plasmid and then treated with AZD8055. As shown in Fig. 2F, increase of CHK1 reduced AZD8055-induced γH2AX and PARP1 cleavage. To ask whether mTOR signaling is required for CHK1 activation by exogenous DNA replication stress, we arrested Rh30 cells in early S phase by exposure to hydroxyurea (HU) for 24 hr and then exposed cells to AZD8055 for a further 24 hr (Fig. 2G). HU induced robust γH2AX, induction of CHK1 and pCHK1-S345 signals, indicating activation of the DNA replication checkpoint. AZD8055 attenuated HU-mediated pCHK1-S345 and the increase of CHK1 by HU. Consistent with these results, AZD8055 enhanced HU-induced γH2AX (Fig. 2H). These data are consistent with the major function of ATR-CHK1 being to enhance DNA damage repair, defects of which lead to DNA replication fork collapse and DNA double strand breaks^4, 5^.

### mTOR Signaling Controls Gene Transcription of *CHK1*

**Figure 3:**
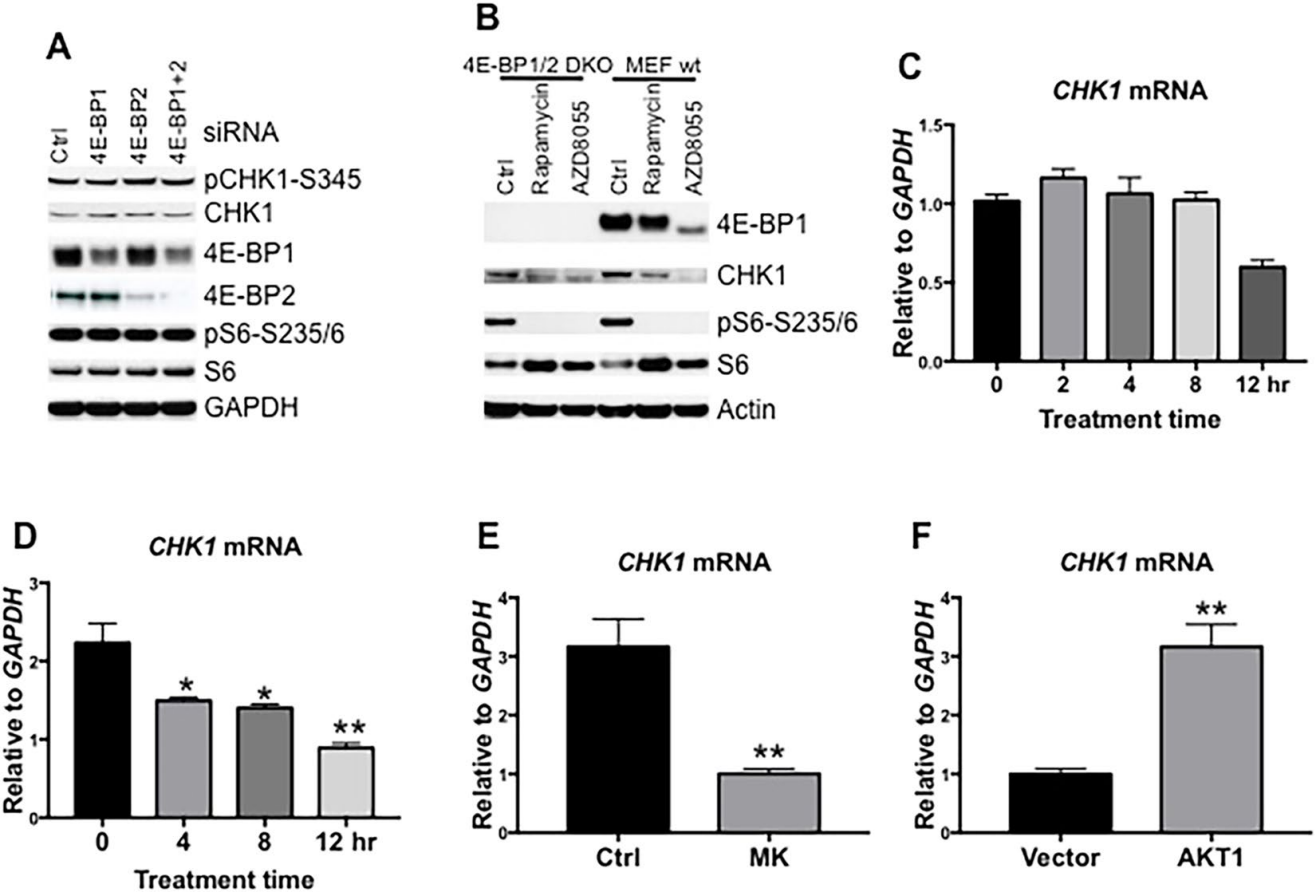
The mTOR Pathway Regulates *CHK1* at Transcription Level. (**A**) Rh30 cells were transfected with control, 4E-BP1, 4E-BP2 and 4E-BP1 plus 4E-BP2 siRNAs. 48 hr later, total proteins were extracted for immunoblotting. (**B**) Wild type (MEF wt) and 4E-BP1/2 DKO MEF cells were treated with rapamycin (100 ng/mL) or AZD8055 (1 μM) for 24 hr. Total proteins were extracted for immunoblotting. (**C**) Rh30 cells were treated with rapamycin (100 ng/ml) for the time indicated. The total RNA was extracted to detect *CHK1* mRNA by real-time RT-PCR with *GAPDH* as internal control. Relative quantity of *CHK1* mRNA was plotted. (**D**) Rh30 cells were treated with AZD8055 (1 μM) for the time indicated. The total RNA was extracted to detect *CHK1* mRNA by real-time RT-PCR with *GAPDH* as internal control. Relative quantity of *CHK1* mRNA was plotted. (**E**) Rh30 cells were treated with MK2206 (MK) for 12 hr. Total RNA was extracted to detect *CHK1* mRNA by real-time RT-PCR with *GAPDH* as internal control. Relative quantity of *CHK1* mRNA was plotted. (**F**) Rh30 cells were transfected with vector or AKT1-wt (AKT1) plasmid. 48 hr later, the total RNA was extracted to detect *CHK1* mRNA by real-time RT-PCR with *GAPDH* as internal control. Relative quantity of *CHK1* mRNA was plotted. Error bars: Mean ± SD (n = 3). *P < 0.05; **P < 0.01. Immunoblots were converted to gray with auto tone by Photoshop program.

A previous study showed that inhibition of mTOR led to a reduced level of CHK1 in HEK293 cells^30^. Our above results showed that inhibition of mTOR leads to decreased CHK1 both in cultured cells and in tumor xenografts. As mTOR signaling has been implicated in both translation and transcription, we next wanted to know the mechanism by which mTOR regulates CHK1 levels. To test whether the AZD8055-mediated reduction of CHK1 is due to the inhibition of cap-dependent protein translation, we downregulated 4E-BP1, 4E-BP2 and 4E-BP1/2 by siRNAs in Rh30 cells and determined protein levels of CHK1. Partial reduction of 4E-BP1/2 did not lead to an increase of CHK1 (Fig. 3A). Similarly, in 4E-BP1/2 double knockout MEF cells (4E-BP1/2 DKO MEFs) that lack functional 4E-BP3^31^, there was no alteration of CHK1 protein level when compared to that of wild type MEF cells, whereas rapamycin slightly and AZD8055 apparently decreased CHK1 in 4E-BP1/2 DKO and wild type MEFs (Fig. 3B). To test whether mTOR signaling may control CHK1 via the S6K branch of the mTORC1 pathway, possibly by regulating *CHK1* transcripts, we determined the mRNA levels of *CHK1* following inhibition of mTOR signaling by real-time RT-PCR. Treatment of Rh30 cells with rapamycin decreased *CHK1* mRNA in 12 hr but without statistical significance (Fig. 3C, P > 0.05). In contrast, AZD8055 induced a progressive decrease in *CHK1* mRNA with statistical significance at 4 and 8 (P < 0.05), and 12 hr (Fig. 3D, P < 0.01). Similarly, MK2206 also significantly decreased *CHK1* mRNA (Fig. 3E, P < 0.01). Conversely, overexpression of *AKT1* significantly increased the mRNA of *CHK1* (Fig. 3F, P < 0.01). Thus mTOR signaling seems to regulate either transcription or transcript stability of *CHK1* mRNA.

### mTOR Signaling Controls Transcription of *CHK1* Gene by Regulating Cyclin Dependent Kinases (CDKs)

**Figure 4:**
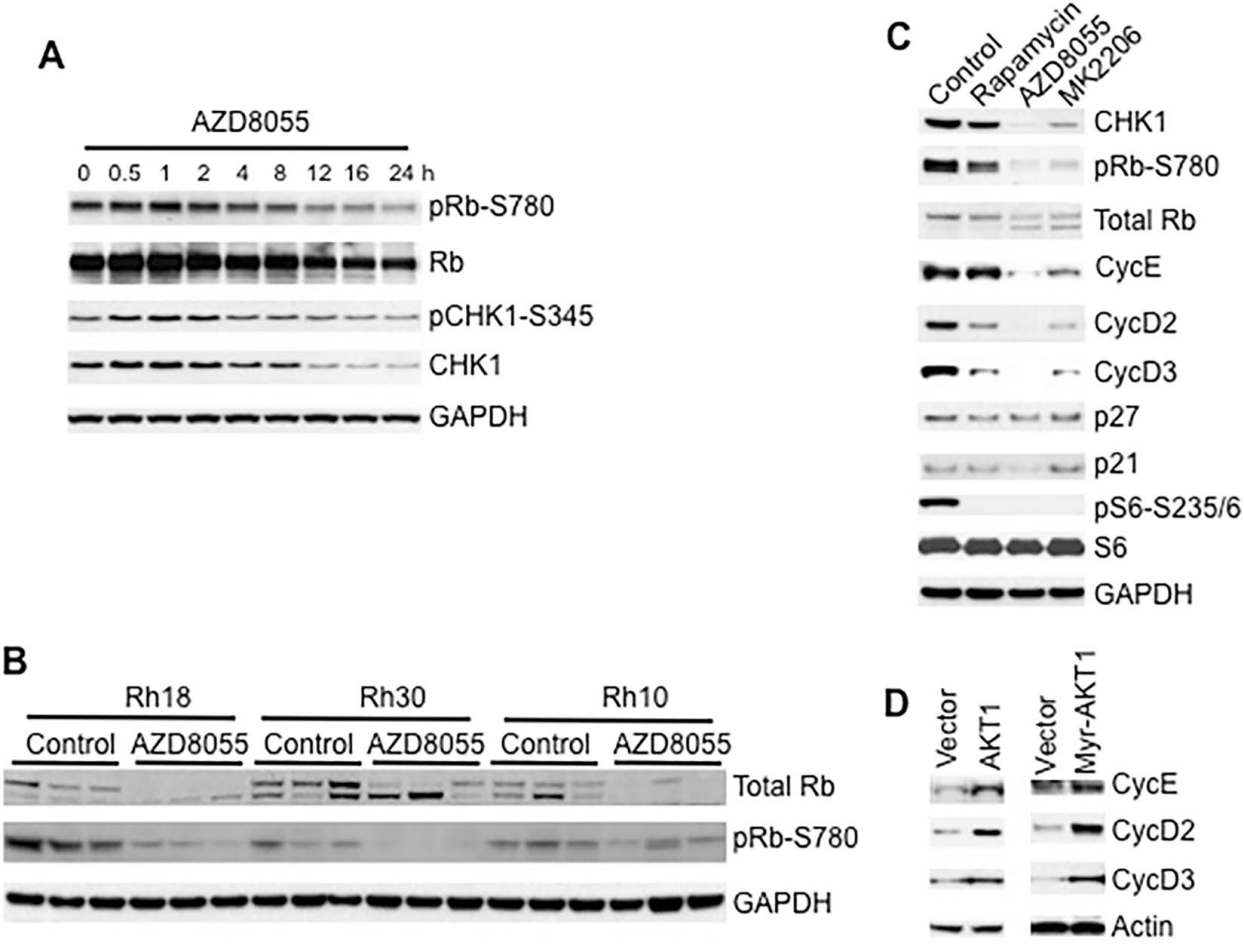
The mTOR Pathway Regulates the Activity of Cyclin D/CDK4/6 and Cyclin E/CDK2. (**A**) Rh30 cells were treated with AZD8055 (1 μM) for the time points as indicated, total proteins were extracted for immunoblotting. (**B**) 24 hr post treatment on day 4, protein extracts of Rh18, Rh30 and Rh10 tumor xenograft models treated with vehicle (Control) or AZD8055 at 20 mg/kg/day were used for immunoblotting to detect the protein levels of Rb and pRb-S780. Three tumors were randomly picked for each group. (**C**) Rh30 cells were treated with rapamycin, AZD8055 or MK2206 for 16 hr. Total proteins were extracted for immunoblotting. (**D**) Rh30 cells were transfected with vector, AKT1 wt, or myr-AKT1 plasmid. 48 hr later, total proteins were extracted for immunoblotting. Immunoblots were converted to gray with auto tone by Photoshop program.

From yeast to mammalian cells, the TOR pathway promotes cell cycle progression by increasing the activity of cyclin dependent kinases (CDKs)^16^. Recently it was found that CDKs are essential for the processing of damaged DNA ends and checkpoint activation^32^. Moreover, *CHK1* is regulated by Rb-E2F1^33^. Our data suggest that mTOR coordinately controls the levels of proteins and transcripts of *CHK1*. In Rh30 cells, AZD8055 gradually reduced CHK1 protein levels with the elongation of treatment time, which was accompanied with gradual decrease of pRb-S780 signal (Fig. 4A), a marker for the activity of CDKs^33^. These observations indicate that mTOR may control the gene expression of *CHK1* by regulating CDKs. To further examine this, we determined the phosphorylation status of Rb both *in vivo* and *in vitro*. In Rh18, Rh30 and Rh10 tumor xenografts, AZD8055 induced dephosphorylation of Rb-S780 (Fig. 4B). Similarly, in cultured Rh30 cells, AZD8055 and MK2206 robustly reduced pRb-S780 whereas rapamycin had a lesser effect (Fig. 4C). mTOR promotes G1 to S phase transition by increasing Cyclin D and E^34^. AZD8055 and MK2206 decreased Cyclin D2, D3 and E in Rh30 cells whereas rapamycin also decreased both Cyclin D2 and D3 but not Cyclin E (Cyclin D2 and Cyclin D3 are the major G1 phase cyclins in rhabdomyosarcoma, Shen and Houghton, unpublished). Of note there were no substantial changes of p21 and p27 levels induced by these agents (Fig. 4C). Conversely, overexpression of AKT1 or myr-AKT1 led to an increase of Cyclin D2, D3 and E (Fig. 4D). Thus mTOR signaling may control transcription of *CHK1* gene by promoting the activity of CDKs.

### Inhibition of CDK4/6 Decreases CHK1 and Induces DNA Damage

**Figure 5:**
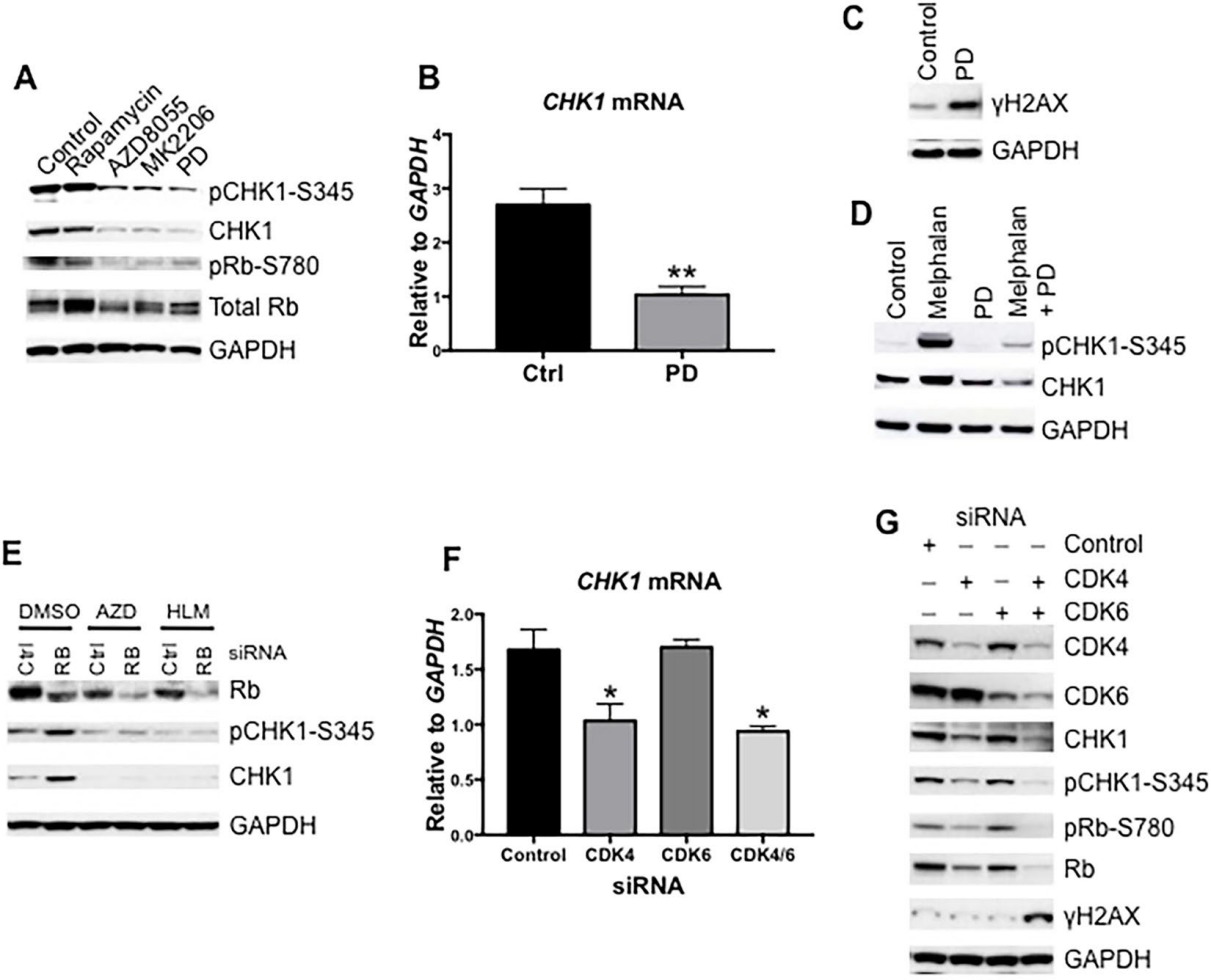
Inhibition of CDK4/6 Decreases CHK1 and Induces DNA Damage. (**A**) Rh30 cells were treated with rapamycin, AZD8055, MK2206 or PD03332991 (1 μM) for 16 hr. Total proteins were extracted for immunoblotting. (**B**) Rh30 cells were treated with PD03332991 for 12 hr. The total RNA was extracted to detect *CHK1* mRNA by real-time RT-PCR with *GAPDH* as internal control. Relative quantity of *CHK1* mRNA was plotted. Error bars: Mean ± SD (n = 3). **P < 0.01. (**C**) Rh30 cells were treated with PD03332991 for 16 hr. Total proteins were extracted for immunoblotting to detect γH2AX. (**D**) Rh30 cells were treated with PD0332991 for 16 hr, then melphalan was added alone or in combination with PD0332991 and incubated for 5 hr. Immunblotting was done to determine CHK1, pCHK1-S345. (**E**) Rh30 cells were transfected with control (Ctrl) or *RB* siRNA. 24 hr later, AZD8055 (AZD, 1 μM), HLM006474 (HLM, 10 μM), or AZD8055 plus HLM006474 (AZD HLM) was added for additional 24 hr. Total proteins were extracted for immunoblotting. (**F**) Rh30 cells were transfected with siRNAs of control, CDK4, or CDK6. 72 hr later, total RNA was extracted to detect *CHK1* mRNA by real-time RT-PCR with *GAPDH* as internal control. Relative quantity of *CHK1* mRNA was plotted. G, Rh30 cells were treated as in Fig. 5F, total proteins were extracted for immunoblotting. GAPDH served as loading controls. Immunoblots were converted to gray with auto tone by Photoshop program.

Our above data suggest that mTOR controls *CHK1* gene transcription by promoting the activity of Cyclin D and E dependent kinases. To test this conjecture, we treated Rh30 cells with rapamycin, AZD8055 or MK2206 in parallel with a CDK4/6 specific inhibitor PD0332991^35, 36^, and determined CHK1 levels by immunoblotting. All the drugs decreased pRb-S780, indicating inhibition of CDKs. Similar to AZD8055 and MK2206, PD0332991 significantly reduced CHK1 and pCHK1-S345 (Fig. 5A). We further analyzed *CHK1* mRNA of Rh30 cells treated with PD0332991. Consistent with the protein levels as shown in Fig. 5A, PD0332991 significantly reduced *CHK1* mRNA (Fig. 5B, P < 0.01), demonstrating that the gene transcription of *CHK1* is indirectly controlled by CDKs. As a consequence, PD0332991 induced γH2AX in Rh30 cells (Fig. 5C). We further treated Rh30 cells with PD0332991, melphalan and PD0332991 plus melphalan and analyzed pCHK1-S345 by immunoblotting. Melphalan induced robust pCHK1-S345 and stabilized CHK1. PD0332991 abolished melphalan-induced pCHK1-S345 that was accompanied with a decrease of total CHK1 protein (Fig. 5D). Moreover, in agreement with the observation that *CHK1* is negatively controlled by Rb-E2F1^37^, partial decrease of Rb by siRNA led to an increase of CHK1 protein and pCHK1-S345 signal (Fig. 5E). In contrast, an E2F specific inhibitor, HLM006474^38^, downregulated CHK1 to levels similar to those following AZD8055 treatment (Fig. 5E). In addition, we found that downregulation of CDK4 but not CDK6 by siRNAs reduced *CHK1* mRNA (Fig. 5F). In agreement, downregulation of CDK4 but not CDK6 resulted in decrease of CHK1 protein, pCHK1-S345, and pRB-S780 (Fig. 5G). Intriguingly, though knockdown of CDK6 alone did not reduce CHK1, it synergized with downgregulation of CDK4 to further decrease pRb-S780, CHK1 protein and pCHK1-S345. Moreover, phosphorylation of H2AX occurred only following knockdown of both CDK4 and CDK6 (Fig. 5G). These results provide additional evidence for the conclusion that the activity of CDKs is required for DNA damage checkpoint activation, and that *CHK1* gene expression is controlled by CDKs^32, 37^. The data suggest that the mTOR pathway controls *CHK1* gene expression, and hence DNA damage checkpoint and DNA repair, by regulating CDKs.

### mTORC1-S6K1 Pathway Controls Cyclin D and E Dependent Kinases

**Figure 6:**
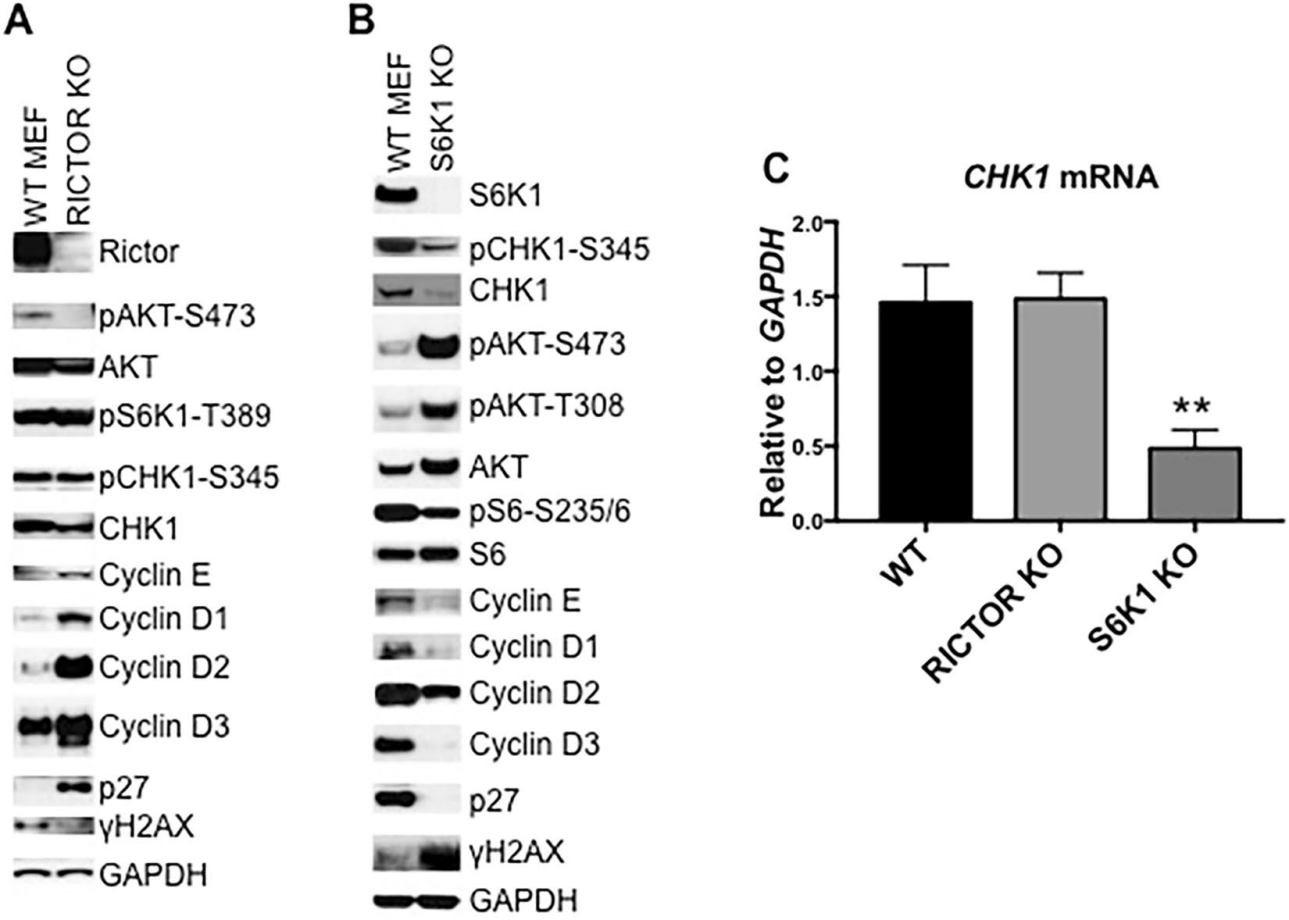
mTORC1-S6K1 Signaling Controls CHK1 via RB-E2F. (**A**) Total proteins of wild type (WT MEF) and RICTOR knockout (RICTOR KO) MEF cells were extracted for immunoblotting. (**B**) Total proteins of wild type (WT MEF) and p70S6K1 knockout (S6K1 KO) MEF cells were extracted for immunoblotting. (**C**) The total mRNA from Wild type (MEF WT), RICTOR KO, and S6K1 KO MEF cells was extracted to detect mouse CHK1 mRNA by real-time RT-PCR with GAPDH as internal control. Relative quantity of CHK1 mRNA was plotted. Error bars: Mean ± SD (n = 3). **P < 0.01. Immunoblots were converted to gray with auto tone by Photoshop program.

PI3K-AKT signaling promotes cell cycle progression by inhibiting p27^34^. Our data demonstrated that inhibition of the mTOR pathway led to decrease of Cyclin D and E but not an increase of p27 (Fig. 4C), suggesting that mTOR kinase controls G1/S phase transition mainly via mTORC1 signaling. To examine this, we determined cell cycle regulators in *RICTOR* knockout mouse embryonic fibroblast cells (Rictor^−/−^ MEF). As shown in Fig. 6A, knockout of *RICTOR* resulted in a slight decrease of the basal level of H2AX phosphorylation, a marginal decrease of CHK1, and elevated levels of cyclins D1, D2, D3 and E. Rictor is required for full activation of AKT and hence suppression of p27. As anticipated, there was no pAKT-S473 signal and a robust increase of p27, but no alteration of pS6K1-T389 in Rictor^−/−^ MEFs. These data indicate that the Rictor-AKT axis promotes but is not essential for the activation and maintenance of mTORC1-S6K1 signaling^39^, and that mTORC1-S6K1 signaling may control CHK1. To test this hypothesis, we examined *S6K1* knockout mouse embryonic fibroblast cells (S6K1^−/−^ MEF). S6K1^−/−^ MEFs demonstrated robust H2AX phosphorylation, significant downregulation of CHK1, and decrease of Cyclins D1, D2, D3 and E. Moreover, depletion of S6K1 led to decreased p27, coincident with the increase of pAKT-S473 and pAKT-T308, indicative of the activation of AKT (Fig. 6B). Consistent with protein levels of CHK1, there was no significant alteration of *CHK1* mRNA in Rictor^−/−^ MEFs (P > 0.05) but significant reduction of *CHK1* mRNA in S6K1^−/−^ MEFs (P < 0.01) when compared with that of wild type MEFs (Fig. 6C). Taken together, these data support the contention that mTOR kinase appears to control CHK1 principally via the mTORC1-S6K1 pathway.

### Inhibition of the mTOR Pathway May Impair the Completion of DNA Replication Following DNA Damage

**Figure 7:**
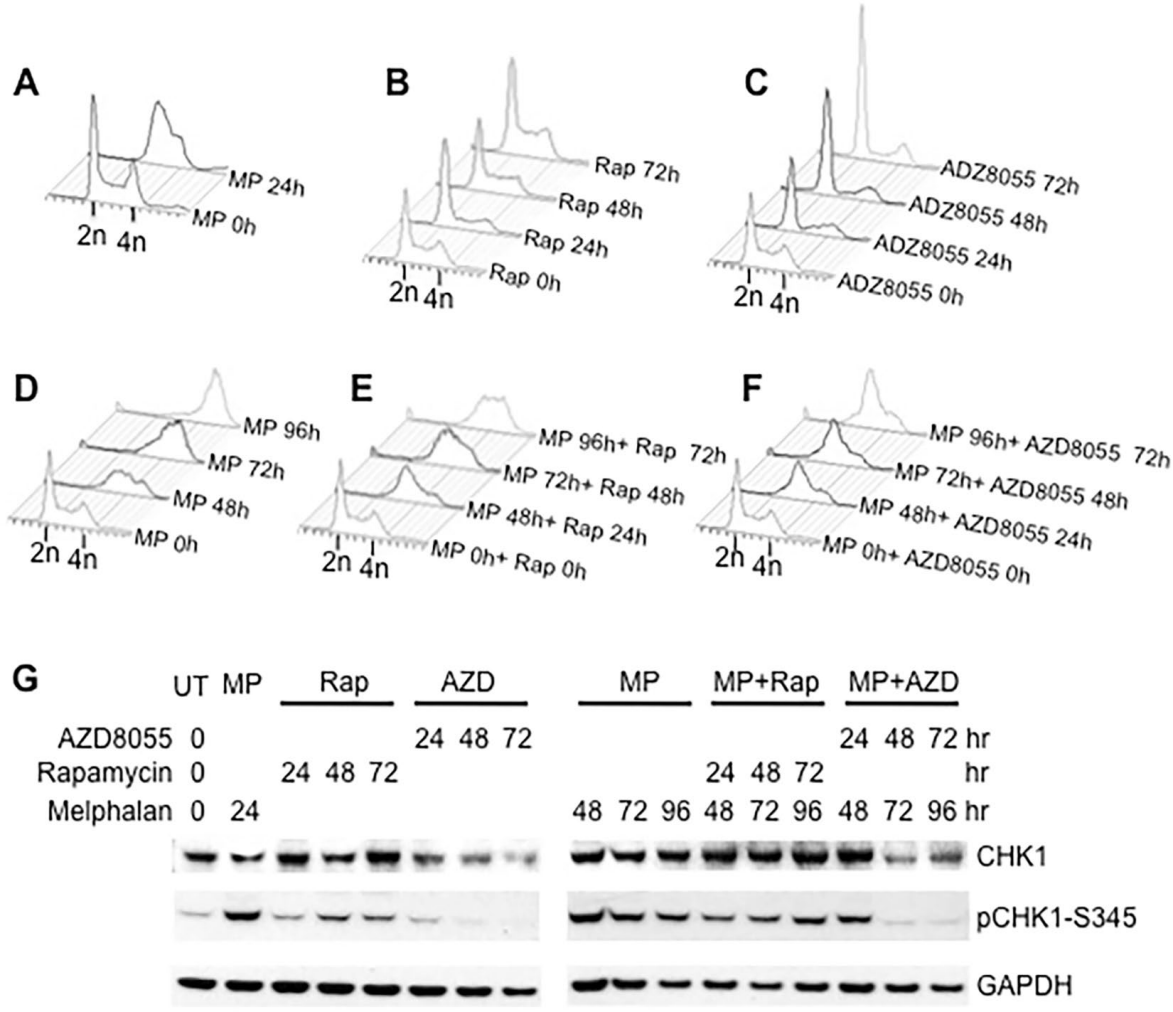
The mTOR Pathway Promotes the S Phase Progression in Response to Mephalan. (**A**) Rh30 cells were arrested in S phase by 2 μg/mL melphalan for 24 hr. (**B**) Rapamycin (100 nM) was added to asynchronous cells for 24, 48 and 72 hr. (**C**) AZD8055 was added to asynchronous cells for 24, 48 and 72 hr. (**D**) Cells were exposed to melphalan for 96 hr. (**E**) cells were arrested by melphalan (24 hr) then exposed to rapamycin for an additional 24, 48 and 72 hr. (**F**) as in (**E**) but cells were exposed to AZD8055 (2 μM). Cell cycle progression was monitored by flowcytometry to show DNA content. 2n, diploid DNA; 4n, tetraploid DNA. MP, melphalan. (**G**) Protein extracts of the same experiments of Fig. 7A–F were used for immunoblotting to detect pCHK1-S345 and CHK1. UT, untreated; MP, melphalan; Rap, rapamycin; AZD, AZD8055. GAPDH served as loading controls. Immunoblots were converted to gray with auto tone by Photoshop program.

The major conserved functions of ATR-CHK1 checkpoint are to prevent the collapse of stalled DNA replication forks and facilitate the completion of DNA replication following DNA damage^3, 4^. Downregulation of CHK1 by mTOR kinase inhibition might result in defects in the slow S phase progression following DNA damage. To examine this, we compared cell cycle progression when cells were treated with rapamycin or AZD8055 in the absence or presence of DNA damage. Cell cycle progression was monitored by flowcytometry to show DNA content and immunoblotting to display CHK1 checkpoint activation. Treatment with the bifunctional alkylating agent, melphalan (24 hr) that induces DNA damage resulted in S phase arrest of most Rh30 cells (Fig. 7A). Treatment of asynchronous (untreated cultures) with rapamycin resulted in G1 accumulation at 24 hr, but cultures became asynchronous at 48 hr (Fig. 7B), suggesting that rapamycin only transiently arrests cell cycle in G1 phase^40^ and then slows down DNA replication afterwards. Rapamycin had little effect on CHK1 and pCHK1 levels (Fig. 7G). In sharp contrast, cells were arrested in G1 phase at 24 hr by AZD8055 and maintained G1 phase arrest even at 72 hr (Fig. 7C). AZD8055 treatment was accompanied with a gradual decrease of CHK1 protein and the basal level of pCHK1-S345 (Fig. 7G). Melphalan treated cells were arrested in S phase in 24 hr (Fig. 7A) but slowly transited S phase and finally arrested in G2/M phase 72 hr later (Fig. 7D). Melphalan induced CHK1 activation at 24 hr. In addition, the pCHK1-S345 signal weakened with the slow transition of S phase by melphalan (Fig. 7G). Rapamycin, added 24 hr after melphalan, further delayed the slow S phase progression induced by melphalan and it took 96 hr for these cells to finish S phase (Fig. 7E). Rapamycin suppressed the effect of melphalan induction of pCHK1-S345, although with time the pCHK1-S345 signal intensified (Fig. 7G). AZD8055 treatment resulted in permanent S phase arrest induced by melphalan (Fig. 7F) and abolished melphalan-induced pCHK1-S345 signals 72 hr after melphalan treatment, with the decrease of CHK1 protein (Fig. 7G). In agreement, there was significantly enhanced phosphorylation of H2AX in cells treated with melphalan in combination with AZD8055 when compared with that treated with melphalan or AZD8055 alone (Supplementary Figure S1). These data indicate that, following DNA damage, mTOR kinase is required for cells to complete DNA replication, and that mTOR signaling promotes the slow S phase progression, at least in part, by maintaining CHK1 levels and hence its activity.

## Discussion

Our recent studies indicate that mTOR signaling may impact responses to DNA damage by regulating FANCD2^41^, and ATM^29^. Here we discovered that inhibition of mTOR kinase activity results in downregulation of CHK1 concomitant phosphorylation of H2AX. In contrast to cells of normal tissues that respond to DNA damage by suppressing mTORC1 signaling, malignant cells have, in most cases, lost the ability to regulate mTORC1 signaling in the presence of DNA damage. Suppression of mTOR signaling by DNA damage involves several checkpoints with p53 as a central component linking the AKT-mTOR and CHK2/CHK1 checkpoint pathways^42^, as DNA damage induced negative regulation of mTORC1 is p53-dependent^8^. Thus, in most cancer cells, the mTOR and p53 pathways are uncoupled. Most cancer cells are deficient in p53 circuitry, either through p53 missense mutations that confer a dominant-negative function, inactivation of the p53 gene by its promoter methylation, mutations that inactivate ARF and overexpression of MDM2^43^. All these aberrations lead to abrogation of p53 function. As a consequence of intrinsic fluctuations in the availability of nutrients, oxygen and growth factors, cancer cells frequently undergo metabolic stress^21, 22^. However, mTORC1 signaling is not suppressed, as demonstrated by maintained phosphorylation of 4E-BP1. Since hypoxia and anoxia promote genome instability^44^, failure to suppress mTORC1 and activate CHK1 may be one of the mechanisms by which cellular stress enhances genome instability of cancer cells.

It was recently reported that mTOR inhibition inhibited DNA damage-induced phosphorylations of CHK1 and reduced the production of CHK1 protein^30^, however the underlying molecular mechanism of the regulation of CHK1 by mTOR remains to be determined. It has been recently reported that CDKs promote DNA damage checkpoint activation and that E2F1-Rb regulates the transcription of *CHK1* ^32, 37^. These findings suggest that maintenance of CHK1 is the common mechanisms by which cell growth signaling pathways including PI3K-AKT and RAS-MAPK, and CDK-Rb-E2F axis promote cell proliferation and survival. Our data show that inhibition of CDKs abolishes melphalan-induced pCHK1 and that inhibition of E2F leads to a loss of CHK1, similar to the effect of inhibiting mTOR by AZD8055, indicating that transcriptional control *CHK1* by mTOR signaling is via enhancing the activity of CDKs. However, although in human rhabomyosarcoma Rh30 cells, knockdown of Rictor by siRNA was more efficient in inducing γH2AX level than knockdown of Raptor by siRNA, in *RICTOR* knockout MEFs, there was no apparent alterations of γH2AX and CHK1. In sharp contrast, S6K1^−/−^ MEFs demonstrated robust H2AX phosphorylation and significant downregulation of CHK1. One of the potential explanations for contradictory results may be the complex feedback signaling of S6K1-IRS and S6K1-mTORC2, and the different nature of transient knockdown of RICTOR by siRNA in Rh30 cells while permanent depletion of RICTOR and S6K1 in MEFs. These results suggest that, though RICTOR is required for the full activation of AKT, the RICTOR-AKT axis might not be essential for the activation and maintenance of mTORC1-S6K1 signaling.

It has been proposed that enhanced spontaneous DNA damage results from oncogenic signaling induced DNA replication stress^13^, although how rapidly proliferating cancer cells with high levels of DNA damage survive genome surveillance systems remains unresolved^13^. The control of the CHK1 checkpoint by mTOR signaling may, at least in part, explain this outstanding enigma. In yeast *Saccharomyces cerevisiae*, the functional ATR-CHK1 homologue, Mec1-Rad53, is responsible for most of the DNA damage response^45^. Similar to the lethal phenotypes of the knockout of either ATR or CHK1, deletion of MEC1 or RAD53 results in cell death. Moreover, MEC1 or RAD53 mutants display high levels of spontaneous DNA damage^46, 47^. Consistent with these results, downregulation of CHK1 by siRNA, inhibition of mTOR, or inhibition of CHK1 kinase resulted in H2AX phosphorylation and PARP1 cleavage. Our data would suggest that in cancer cells where mTOR activity is not suppressed by DNA damage, CHK1 is maintained and may contribute to enhanced survival. In contrast, where mTOR signaling is inhibited in response to DNA damage, for example in proliferating cells of normal tissues, CHK1 protein would be lost, concomitant with extensive DNA damage, leading to engagement of cell death programs. This is consistent with the idea that if damage is not repaired in a specific window of time, cell death is initiated, thus preventing inheritance of genomic errors.

In many cancers the PI3K-AKT and RAS-MAPK signaling pathways are deregulated and both pathways converge on mTOR signaling. It has been well established that cancer depends on these two central growth-promoting signaling pathways for survival^34^. The control of *CHK1* and hence CHK1 checkpoint activity by mTOR kinase in cancer cells may, in part, explain the “addiction” of cancers to PI3K-ATK-mTOR signaling^48, 49^. In addition, most cancer cells have acquired the ability to deregulate cell cycle checkpoints to increase the overall activity of CDKs^12^.

**Figure 8:**
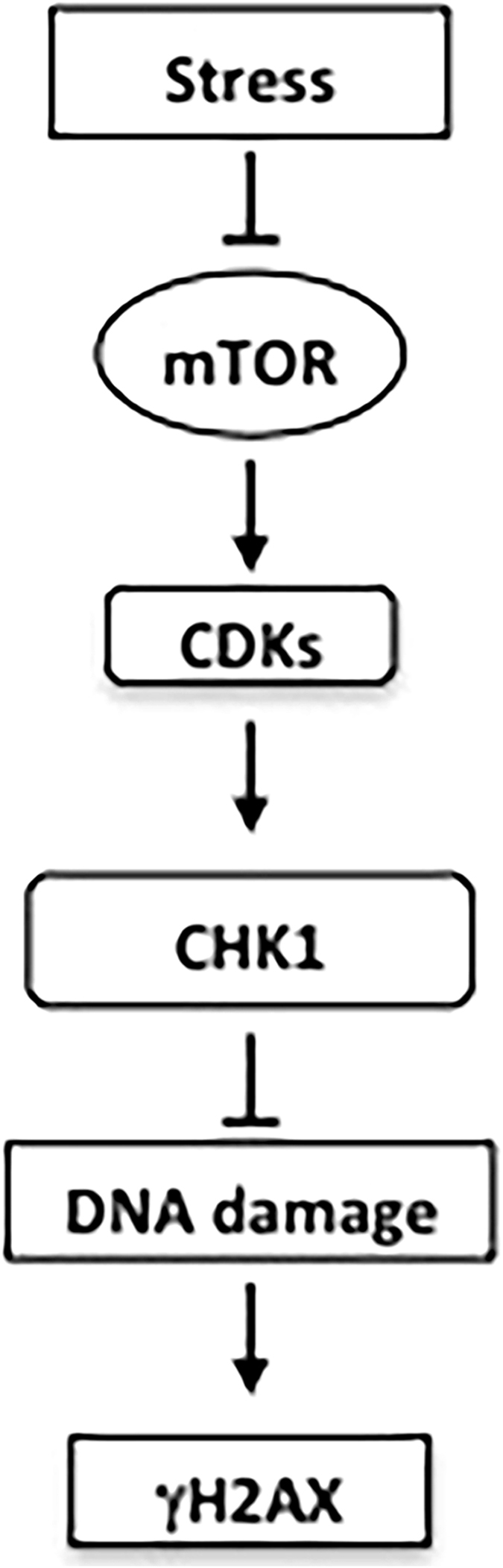
A scheme showing the functional linkage among the mTOR pathway, metabolic stress and DNA damage response by CHK1.

In summary, we found a conserved functional linkage between the mTOR pathways and DNA damage response using pharmacologic and genetic approaches. Our results suggest that the mTOR pathway may link metabolic stress with DNA damage responses to control cell cycle progression and survival in response to the ever-changing availability of nutrients, growth factors and oxygen from the microenviroment of cancer cells that lead to DNA damage (Fig. 8).

## Methods

### Drugs and Reagents

Rapamycin was from the NCI drug repository. Melphalan and hydroxyurea were purchased from Sigma (St. Louis, MO). AZD8055, MK2206, PD0332991 and AZD7762 were from Selleck Chemicals (Houston, TX). HLM006474 was from EMD Millipore (Billerica, MA).

### Solid Tumor Xenografts Studies

CB17SC scid^−/−^ female mice (Taconic Farms, Germantown, NY) were used to propagate subcutaneously implanted rhabdomyosarcoma. All mice were maintained under barrier conditions and experiments were conducted using protocols and conditions approved by the institutional animal care and use committee of The Ohio State University (IACUC protocol 2010A00000192). This study was approved by the ethic committee of The Ohio State University. AZD8055 was administered P.O. daily at 20 mg/kg per day. Rapamycin was dissolved in DMSO (5% final concentration) and diluted in 5% Tween-80 in water and administered I.P. daily at a dose of 5 mg/kg. Tumors were harvested post treatment on day 1 or day 4 at different time points.

### Cells, siRNA and Plasmids

Rhabdomyosarcoma Rh30 cells were cultured in RMPI 1640 (GIBCO) supplemented with 10% heat-inactivated FBS (GIBCO). Control and ON-TARGETplusSMARTpool siRNAs of CHK1, mTOR, RB, CDK4, CDK4, RAPTOR, RICTOR, 4E-BP1 and 4E-BP2 were purchased from Dharmacon (Chicago, IL). Plasmid pCMV-Akt1-wt was from Said Sebti. Plasmid pcDNA4-CHK1-Flag was provided by Keziban Unsal-Kacmaz. Plasmid pcDNA3-Myr-AKT1 was a gift of William Sellers (Addgene plasmid 9008). S6K1 knockout MEF was provided by George Thomas. RICTOR knockout MEFs were provided by Mark A. Magnuson. 4E-BP1/2 double knock-out MEFs were from Nahum Sonenberg. MEF cells were cultured in DMEM (GIBCO) supplemented with 10% heat-inactivated FBS (GIBCO). Lipofectamine 2000 was from Invitrogen (Carlsbad, CA) and transfection of siRNA or plasmids in Rh30 cells was performed according to the manufacture’s instructions.

### Immunoblotting and Flowcytometry

Cells were lysed on ice in RIPA lysis buffer (Cell Signaling Technology) supplemented with protease inhibitors and phosphatase inhibitor (Roche), and 1 mM PMSF (Sigma). Immunoblots were probed with the following antibodies: S6, pS6 (S235/236), AKT, pAKT (S473, T308), CHK1, pCHK1 (S345), CHK2, pCHK2 (T68), mTOR, RICTOR, RAPTOR, S6K1, pS6K1 (T89), GSK3β, pGSK3β (S9), p4E-BP1 (T37/46), β-Actin, cyclin E, cyclin D1, cyclin D2, cyclin D3, p21, p27, GAPDH, Rb, pRb (S780) (Cell Signaling Technology); pH2AX (S139) (Upstate); PARP1, cleaved PARP1 (Abnova); CDK4, CDK6 (Santa Cruz); For cell cycle analysis, cells were trypsinized following treatment, fixed with 70% ethanol and stored at 4 °C for subsequent FACS analysis of DNA content.

### RNA Isolation, cDNA Synthesis and RT-PCR

Total RNA from cultured cells or mouse tumor xenografts were extracted with mirVana miRNA Isolation Kit (Ambion) according to the total RNA isolation protocol. Reverse transcription were performed using the High Capacity RNA-to-cDNA kit according to the manufacturer’s instructions. Real-time PCR was performed on the 7900HT Fast Real-Time PCR System using the TaqMan® Universal Mastermix II. Human and mouse *CHK1* expression was quantified in real-time with *CHK1* specific FAM dye-labeled MGB-probes and normalized to *GAPDH* or beta-ACTIN (Applied Biosystems).

### Statistical Analyses

Graphs were constructed using GraphPad Prism (Graphpad Software, San Diego, CA). All data are presented as mean ± SEM. Statistical significance was determined by unpaired two-tailed *t* tests or two-way ANOVAs. *P* < 0.05 was considered statistically significant.

## Electronic supplementary material

Supplementary Information
