# Supplementary material for: Regulation of CHK1 by mTOR contributes to the evasion of DNA damage barrier of cancer cells
Source: Sci Rep. 2017 May 8;7:1535. doi: 10.1038/s41598-017-01729-w (PMC5431544; doi:10.1038/s41598-017-01729-w)
Supplement: Supplementary file 1 — Supplementary Information [file 41598_2017_1729_MOESM1_ESM.pdf]

Supplementary Information

Regulation of CHK1 by mTOR contributes to the evasion of DNA damage barrier of cancer cells

Xinhui Zhou, Weijin Liu, Xing Hu, Adrienne Dorrance, Ramiro Garzon, Peter J. Houghton and Changxian Shen

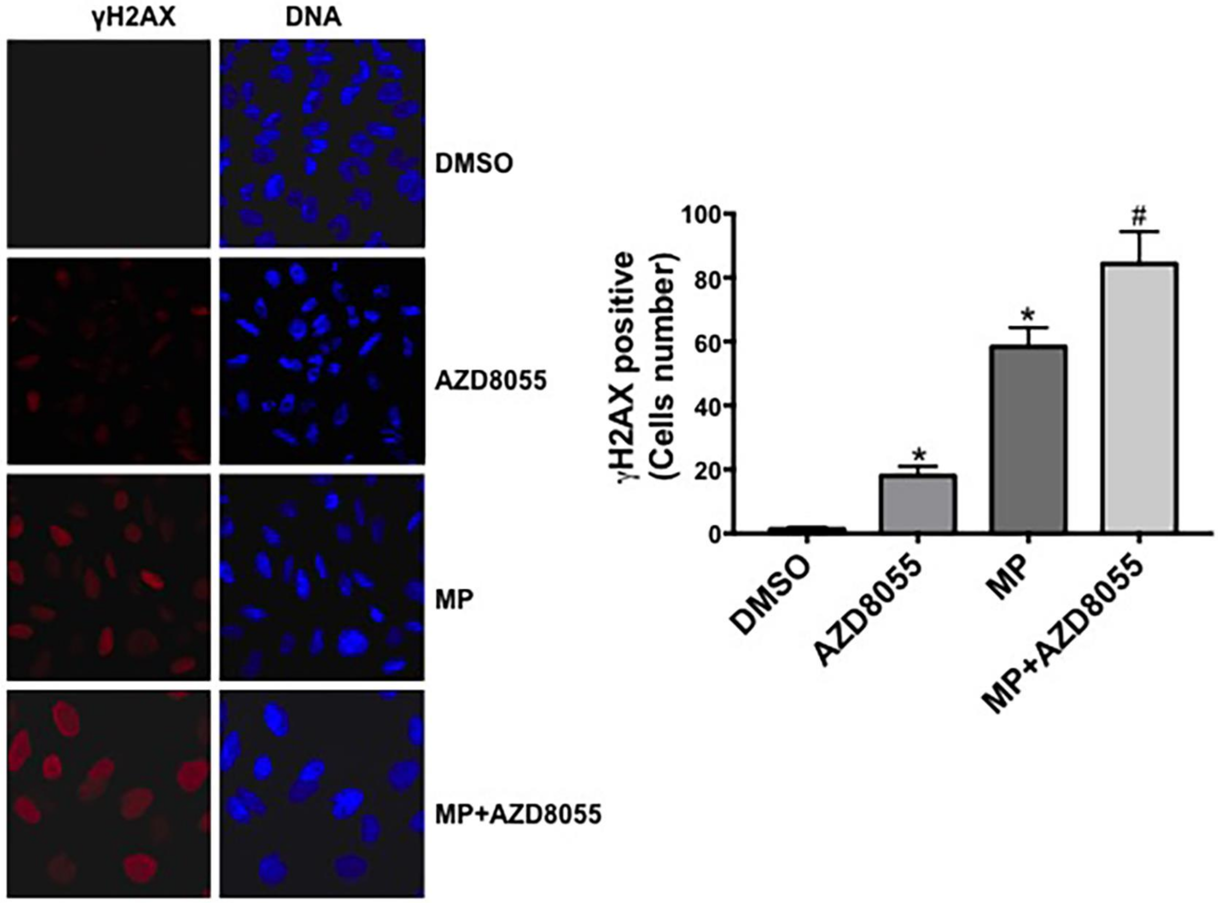

Supplementary Figure S1. Inhibition of mTOR Signaling Results in Spontaneous DNA Damage and Enhances Melphalan-mediated Phosphorylation of H2AX. Rh30 cells were treated with DMSO as control, AZD8055 (2 μM) for 72hr, melphalan (2 μg/mL) for 96 hr, or melphalan for 24 hr followed by addition of AZD8055 for additional 72 hr. After treatment, cells were subjected to immunofluorescence analysis of γH2AX with a rabbit anti-γH2AX antibody and Alexa Fluor 555 donkey anti-rabbit secondary antibody. DAPI staining was used to stain the nuclei. Magnification 40x. MP, melphalan. γH2AX positive cells were counted in a total of 100 cells in each sample with triplicate. *$p<0.01$ vs DMSO; #$p<0.05$ vs AZD8055 or MP+AZD8055.

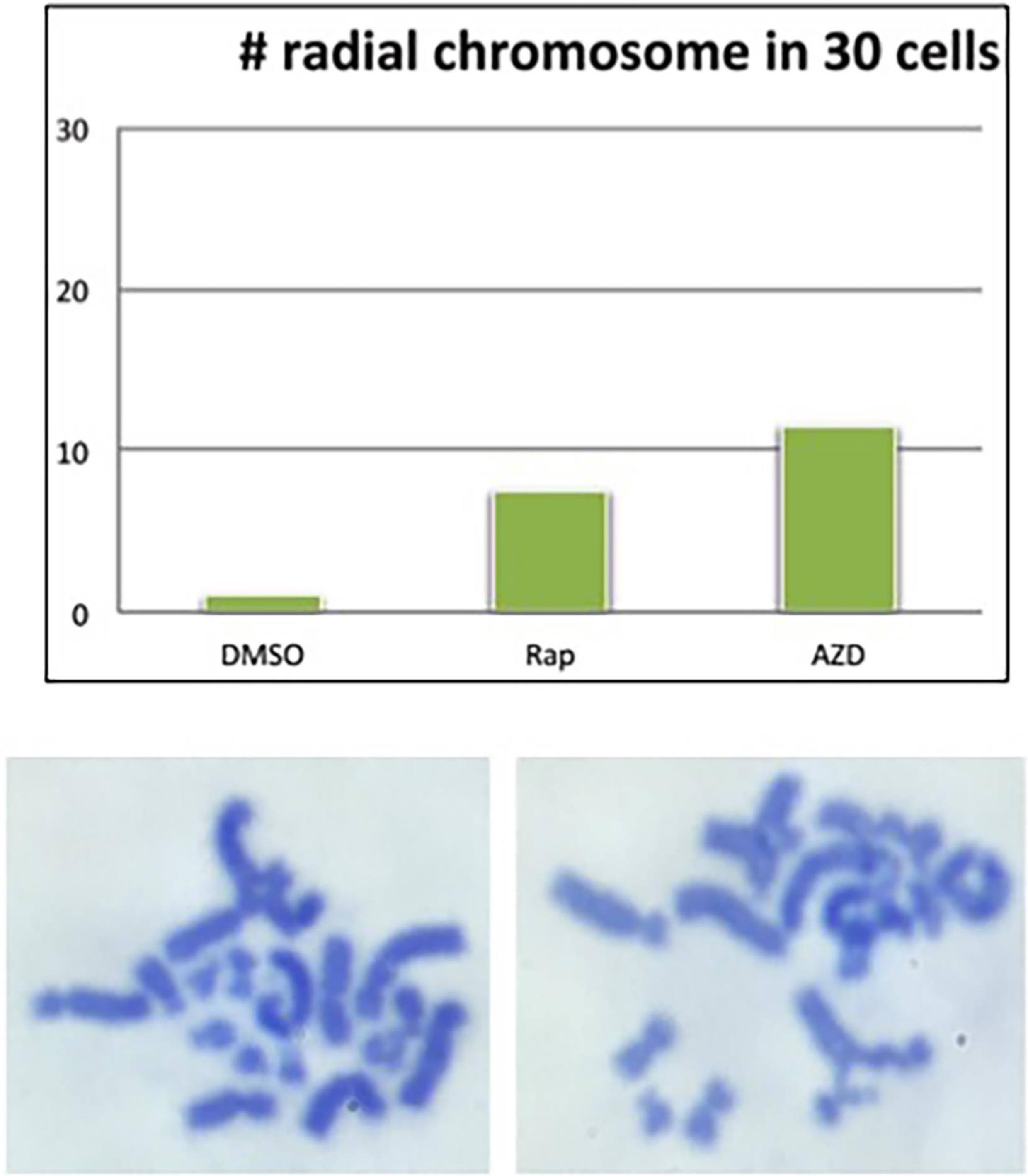

Supplementary Figure S2. Inhibition of mTOR Signaling Leads to DNA damage. Rh30 cells were treated with DMSO as control, rapamycin (100 nM), or AZD8055 (2 μM) for 24hr. After treatment, the cells were subjected to chromosome spreading analysis of metaphase chromosomes. 30 cells in metaphase were analyzed in each treatment group. The upper panel shows the number radial chromosomes in a total of 30 metaphase cells in each group. The low

panel shows two representative metaphase cells with radial chromosomes after AZD8055 treatment. Rap, rapamycin; AZD, AZD8055.
